# Which residual symptoms predict relapse after successful electroconvulsive therapy for late-life depression?

**DOI:** 10.1192/j.eurpsy.2022.460

**Published:** 2022-09-01

**Authors:** S. Lambrichts, K. Vansteelandt, M. Wagenmakers, M. Oudega, J. Obbels, A. Dols, F. Bouckaert, P. Sienaert

**Affiliations:** 1 KU Leuven, University Psychiatric Center Ku Leuven, Kortenberg, Belgium; 2 Amsterdam University Medical Center, Amsterdam Neuroscience, Amsterdam, Netherlands

**Keywords:** Electroconvulsive therapy, Residual symptoms, Relapse, Late-life depression

## Abstract

**Introduction:**

*Introduction*: Residual depressive symptoms are common after a successful acute treatment of late-life depression (LLD), and their presence predicts increased risk of relapse. While electroconvulsive therapy (ECT) is the most effective treatment for LLD, little is known about which particular symptoms remain and impact long-term outcome after a successful acute ECT course.

**Objectives:**

*Objectives*: We aimed to assess the association between specific residual depressive symptoms after an effective acute ECT course for LLD and relapse at six-month follow-up.

**Methods:**

*Methods*: In this prospective cohort study, including 110 patients aged 55 years and older with LLD, information about relapse was collected six months after the acute ECT course. Relapse was defined as a Montgomery-Åsberg Depression Rating Scale (MADRS) score >15, hospital admission or restart of ECT. We used multivariable stepwise logistic regression models including the scores on the 10 individual MADRS items at the end of the acute ECT course to predict relapse.

**Results:**

*Results*: Of the 80 responders with available six-month follow-up data, 29 patients (36.25%) had suffered relapse. Higher scores on the MADRS items ‘reduced sleep’ (odds ratio (OR)=2.03, 95% confidence interval (CI)=1.11-3.69, p=0.0214) and ‘lassitude’ (OR=1.62, 95% CI=1.00-2.62, p=0.0497) at the end of the acute ECT course were significantly associated with increased risk of relapse at six-month follow-up.

**Conclusions:**

*Conclusions*: Some residual depressive symptoms, including sleep disturbance and fatigue, may help better identify patients vulnerable to relapse following a successful acute ECT course for LLD. Future studies assessing interventions that target specific residual symptoms may further reduce post-ECT depressive relapse.

**Disclosure:**

No significant relationships.

